# A250 ESOPHAGOGASTRIC JUNCTION OUTFLOW OBSTRUCTION SECONDARY TO METASTATIC SQUAMOUS CELL CARCINOMA A CASE REPORT AND REVIEW OF THE LITERATURE

**DOI:** 10.1093/jcag/gwae059.250

**Published:** 2025-02-10

**Authors:** Z Alhashimalsayed, K McIntosh

**Affiliations:** Gastroenterology, Western University, London, ON, Canada; Gastroenterology, Western University, London, ON, Canada

## Abstract

**Background:**

Esophagogastric junction outflow obstruction (EGJOO) is a condition characterized by impaired relaxation of the lower esophageal sphincter, with intact peristalsis. The Chicago Classification version 4.0 (CCv4.0) updated the diagnostic criteria to include not only elevated integrated relaxation pressure (IRP) but also dysphagia, high intrabolus pressure, and abnormal findings on timed barium esophagram or FLIP. EGJOO can be caused by secondary factors such as structural issues, medications, inflammatory conditions, or cancer, with rare cases linked to metastatic disease, like SCC of the skin. Identifying metastatic cancer as a potential cause in motility disorders requires careful evaluation in unusual cases

**Aims:**

A 63-year-old man with a two-month history of dysphagia, 20-pound weight loss, chest pressure, and regurgitation did not respond to PPIs. His medical history included type 2 diabetes, two renal transplants, and a past cutaneous squamous cell carcinoma (SCC) treated with surgery and radiotherapy. He was on long-term immunosuppressive therapy. Endoscopy showed a tightly constricted LES, and HRM confirmed EGJOO with impaired LES relaxation. CT imaging revealed an 8 cm mass at the gastroesophageal junction and a 4 cm lung lesion. Biopsy confirmed metastatic SCC, indicating recurrence. This case emphasizes the need to consider malignancy in EGJOO, especially in patients with cancer history and immunosuppression

**Methods:**

N

**Results:**

EGJOO secondary to metastatic SCC is rare but important to recognize. Diagnostic criteria in CCv4.0 help reduce false positives by incorporating symptoms and confirmatory tests, differentiating clinically significant EGJOO from incidental findings. It is crucial to systematically evaluate for secondary causes, particularly malignancy, though routine imaging may not provide additional benefits over endoscopy and timed barium esophagram unless there is a strong suspicion

This case highlights how a history of SCC and long-term immunosuppression can contribute to metastasis to the GEJ. Management primarily focuses on palliative care to relieve dysphagia and enhance quality of life, using endoscopic, pharmacological, or radiotherapy options, while more invasive treatments like POEM or pneumatic dilation may be less appropriate for cancer patients

**Conclusions:**

This case report highlights a rare cause of EGJOO secondary to metastatic SCC, emphasizing the need for thorough evaluation in dysphagia cases. Findings suggest that cancer should be considered in EGJOO patients with known cancer histories or immunosuppression. Early recognition and palliative management can significantly enhance the patient’s quality of life by effectively addressing dysphagia. This report contributes to the understanding of EGJOO as a heterogeneous condition with various underlying pathologies, including metastatic cancer

**Funding Agencies:**

None

